# Highly Pathogenic Avian Influenza A(H5N1) Virus Infection in Cats, South Korea, 2023

**DOI:** 10.3201/eid3012.240154

**Published:** 2024-12

**Authors:** Yong-Myung Kang, Gyeong-Beom Heo, Se-Hee An, Hyunho Lee, Eunhye Park, Ra Mi Cha, Yun Yueng Jang, Mingeun Sagong, Ah-Young Kim, Jongho Kim, Eun-Kyoung Lee, Seong Hee Kim, Kyungki Lee, Bokkyung Ku, Youn-Jeong Lee, Kyunghyun Lee, Kwang-Nyeong Lee

**Affiliations:** Animal and Plant Quarantine Agency, Gimcheon-si, South Korea (Y.-M. Kang, G.-B. Heo, S.-H. An, R.M. Cha, Y.Y. Jang, M. Sagong, A.-Y. Kim, J. Kim, E.-K. Lee, S.H. Kim, Kyungki Lee, B. Ku, Y.-J. Lee, Kyunghyun Lee, K.-N. Lee); Kyungpook National University, Daegu, South Korea (Y.-M. Kang); Seoul National University, Seoul, South Korea (G.-B. Heo); Seoul Metropolitan Government Research Institute of Public Health and Environment, Gwacheon-si, South Korea (H. Lee, E. Park)

**Keywords:** influenza, viruses, highly pathogenic avian influenza, H5N1, cat, raw duck meat, South Korea, zoonoses

## Abstract

In July 2023, cases of highly pathogenic avian influenza (HPAI) were reported at 2 shelters for stray cats in Seoul, South Korea. The cause of infection was suspected to be improperly sterilized raw food made from domestic duck meat, which was manufactured in South Korea. All viruses isolated from cats at the shelters and from the raw food belonged to HPAI A(H5N1) clade 2.3.4.4b. The gene constellation of all viruses was most similar to that of viruses isolated in Korea in November 2022. Of note, the viruses isolated from infected cats harbored mutations E627K or D701N in polymerase basic 2, which are indicative of adaptation to mammals. Postmortem examination revealed systemic pathologic lesions and the presence of widespread virus in different tissues. Thus, consumption of raw duck meat contaminated with HPAI virus likely caused systemic symptoms and death in cats, indicating the introduction of mammal-adapted mutations of the virus.

Highly pathogenic avian influenza virus (HPAIV) (H5Nx) subtype descendents of the H5N1 Goose-Guangdong (Gs/Gd) lineage emerged in 1996; since then, derivatives of H5Nx have disseminated intercontinentally through wild migratory waterfowl and human activity (*1*–*3*). Since 2020, HPAI H5 viruses belonging to the Gs/Gd lineage have become panzootic, demonstrating continual reassortment with low pathogenicity avian influenza viruses (LPAIV). Those viruses have shown unprecedented global spread among poultry and wild birds, even infecting mammals and humans (*1*,*4*). In most cases, mammal infection results from direct or indirect contact with infected birds or from consumption of dead birds, suggesting that the avian virus can be transmitted to mammal hosts (*5*).

Viruses belonging to H5Nx clade 2.3.4.4b caused major outbreaks in wild birds in Asia, Europe, Africa, and America (*6*,*7*); infections even extended to both terrestrial and aquatic mammals (*8*). Wild mammals, such as red foxes, lynxes, and skunks, and domestic mammals, including pet ferrets, domestic mink, raccoon dogs, and arctic foxes, have been infected by H5N1 clade 2.3.4.4b viruses; moreover, the disease has been detected in aquatic mammal species including seals and sea lions in North and South America (*6*,*9**,**10*). In addition, a report from Italy in July 2023 revealed infected cats and dogs living in close proximity to humans (*10*); studies conducted since June 2023 report that 29 domestic cats in at least 6 regions of Poland were infected with the H5N1 clade 2.3.4.4b virus (*10*–*12*).

In South Korea, HPAIV infection of cats and dogs on poultry farms affected by HPAIV were reported in 2015 and 2016. Those infections are thought to have occurred through close contact with, or consumption of, HPAIV-infected birds (*13*,*14*). HPAIV was later confirmed in cats at 2 different cat shelters in 2023. In July 2023, cats housed in a cat shelter in Seoul were found dead, leading the owner to request diagnostic tests at a private diagnostic institution. Subsequently, a veterinary laboratory at Seoul National University (SNU) became involved in the diagnosis of those samples. The SNU laboratory contacted the Animal and Plant Quarantine Agency (APQA) in South Korea with the HPAIV-positive test results; the HPAIVs were isolated from dead cats and belonged to H5N1 clade 2.3.4.4b with a D701N mutation in the polymerase basic (PB) 2 gene (*15*). A second shelter in Seoul reported suspected clinical signs of HPAIV infection in cats 5 days later. We obtained additional clinical samples from cats and the environments of the 2 shelters and conducted various tests to identify the source of infection and to characterize the viruses.

In this study, we describe identifying the source of infection through environmental sampling, epidemiologic investigations, and genetic analysis. We also evaluate the risk for human infection and transmission between mammals. Furthermore, we explore disease pathogenesis, focusing on virus replication in tissues and associated pathologic sequelae.

## Materials and Methods

### Case Description and Sampling

On July 24, 2023, SNU contacted the APQA, the national reference institute headquarters responsible for avian influenza diagnosis in South Korea, to report possible HPAIV infection of cats housed in a shelter located in Yongsan-gu, Seoul (shelter 1). To ensure the results and investigate the case, we collected additional samples from 3 frozen cat carcasses and 2 live cats at the shelter; we also collected blood from 2 live cats, neither of which showed clinical signs for serologic testing. On July 29, 2023, a vet charged with examining hospitalized cats from another cat shelter in Gwanak-gu, Seoul (shelter 2), also reported suspected cases of HPAIV infection. A cat carcass, as well as nasal swab samples from 4 sick cats housed at the animal hospital and from the 122 cats remaining at shelter 2, were collected and tested by the Seoul local veterinary service. The clinical samples and the cat carcasses that tested positive for the H5 gene from shelter 2 were sent to APQA. We performed postmortem examination on all the cat carcasses sent to APQA from the 2 shelters and collected tissue samples from various organs. All those samples and other clinical swab samples were subjected to molecular diagnostic tests for avian influenza or other suspected diseases.

To determine the location and the extent of contamination of the shelters, we conducted environmental sampling (183 swabs) at various locations within 2 shelters within 2–3 days of the report of HPAIV. The swabs were placed in a viral transport medium and delivered to the APQA directly. We categorized environmental samples according to sampling location, type of object sampled, the structures within the shelter itself, whether the items were cat-related (e.g., food), and samples from wildlife residues located outside the shelter.

Furthermore, we conducted epidemiologic environmental sampling at relevant sites associated with contaminated raw food, such as raw duck meat food manufacturers, slaughterhouses, meat storage facilities, suspected duck farms, other cat shelters, and locations associated with feral cats. We collected 214 samples from 9 different locations and tested them at the APQA. In addition, we also sampled and tested all types of raw food for cats or dogs on the market (produced by 10 other manufacturers); in total, we tested 65 raw food products and 24 raw meats of duck or chicken origin.

### Molecular Detection, Isolation, and Sequencing

We tested swab samples, tissue samples, and virus isolates by real-time reverse transcription PCR (RT-PCR), as described previously (*16*,*17*), to detect the matrix (M), H5, or H7 genes of avian influenza (*18*). If a sample tested positive for H5, we amplified the cleavage site within the H5 hemagglutinin (HA) gene by RT-PCR and determined nucleotide sequences using an Applied Biosystems ABI 3500xL Genetic Analyzer (ThermoFisher Scientific, https://ww.thermofisher.com) for pathotyping (*18*).

To isolate and characterize the virus, we inoculated H5-positive samples into 10-day-old embryonated chicken eggs and incubated for 48 hours at 37°C. We extracted viral RNA from the infectious allantoic fluid using an NX-48 Viral NA kit (Genolution, https://genolution.co.kr). We amplified all 8 segments of the isolates by RT-PCR (*19*). We performed complete-genome sequencing using the Illumina Miseq platform with the Nextera DNA Flex Library Prep Kit (https://www.illumina.com) and assembled genomic sequences by using CLC Genomics Workbench version 23 (QIAGEN, https://www.qiagen.com). We deposited the nucleotide sequences of 12 viruses isolated in this study in the GISAID database (https://www.gisaid.org; accession nos. EPI_ISL_18819799–EPI_ISL_18819810). We downloaded the reference datasets for phylogenetic analysis of all the gene segments characterized in this study from GenBank and the GISAID EpiFlu Databases. We aligned those sequences with MAFFT (https://mafft.cbrc.jp/alignment/software) using the default parameters for FASTA alignment. We removed all untranslated regions and retained only the protein-coding sequences of each segment. We constructed maximum-likelihood trees based on the aligned sequences using RAxML on XSEDE version 8.2.12 (*20*). We used bootstrap analysis with 1,000 replicates to assess the reliability of the trees and generated the tree displays by using the interactive Tree of Life program (*21*).

### Pathologic Examination

We conducted necropsy to confirm HPAIV diagnosis and to examine pathologic lesions. We collected tissue samples (brain, heart, lung, spleen, kidney, liver, pancreas, and intestine), fixed for 24 hours in 10% buffered neutral formalin, and then processed for paraffin embedding. We then cut 4-µm sections, mounted, dewaxed, and stained with hematoxylin and eosin. We analyzed duplicate sections by immunohistochemistry to determine the distribution of influenza virus antigens using a monoclonal antibody specific for influenza A virus nucleoprotein (Bio-Rad Laboratories, https://www.bio-rad.com). We used a biotinylated goat anti-mouse IgG and an avidin-biotin complex system, using the RedMap Kit (all Roche, https://www.roche.com) as the chromogenic substrate. We incubated the negative control slide in phosphate-buffered saline instead of the primary antibody.

### Serologic Testing

We treated serum samples from 2 surviving cats from shelter 1 with a receptor-destroying enzyme (Denka Seiken, https://www.denka.co.jp), inactivated at 56°C for 30 minutes, and chilled at 10°C. We performed hemagglutination inhibition assays using standard methods and homologous antigens (*22*).

## Results

### Detecting, Isolating, and Characterizing Viruses from Cats in the Shelters

We detected the H5 gene in nasal swab samples and all tested organs from the 3 carcasses at shelter 1 and 1 cat carcass from the shelter 2–related animal hospital (Table 1). In addition, nasal swab samples from the 3 living cats at the animal hospital and its related shelter 2, were positive for the H5 gene (Table 1). All H5-positive samples had multiple basic amino acid residues at the cleavage site of the HA gene (PLREKRRKR/G), corresponding to the motif that denotes the HPAI virus, was detected. In addition, the analysis of the neuraminidase (NA) sequences assigned all the viruses sequenced to the N1 subtype. As expected, HPAI H5N1 viruses were isolated from the affected cats from the 2 shelters. The 2 surviving cats in shelter 1 had H5-specific antibodies (hemagglutination inhibition titer, 2^5^) (Table 1).

**Table 1:** Real-time RT-PCR results for the H5 gene in study of highly pathogenic avian influenza virus A(H5N1) infection in cats, South Korea, 2023*

Location	ID	Clinical signs	Cycle threshold value										
			Brain	Feces	Heart	Intestine	Kidney	Liver	Lung	Lymph node	Nasal swab	Spleen	Trachea
Yongsan-gu													
Shelter 1	Cat carcass no. 1	Death	33	32	32	27	32	27	21	28	35	26	26
	Cat carcass no. 2	Death	20	28	21	27	25	15	18	22	27	21	26
	Cat carcass no. 3	Death	28	31	25	27	27	17	19	24	36	25	23
	Cats nos. 4–5	None	NT	NT	NT	NT	NT	NT	NT	NT	–†	NT	NT
Gwanak-gu													
Animal hospital	Cat carcass no. 1	Death	22	27	20	19	18	12	15	21	19	17	24
	Cat no. 1	Severe	NT	NT	NT	NT	NT	NT	NT	NT	35	NT	NT
	Cats nos. 2–4	Moderate	NT	NT	NT	NT	NT	NT	NT	NT	–‡	NT	NT
Shelter 2	Cat no. 5	None	NT	NT	NT	NT	NT	NT	NT	NT	29	NT	NT
	Cat no. 6	None	NT	NT	NT	NT	NT	NT	NT	NT	24	NT	NT
	Cats nos. 7–126	None	NT	NT	NT	NT	NT	NT	NT	NT	–§	NT	NT

*ID, identification number; NT, not tested; RT-PCR, reverse transcription PCR; –, negative. †Two live cats from shelter 1 were negative on H5 real-time RT-PCR but showed seroconversion against H5 antigens. ‡Three cats from the animal hospital were negative on H5 real-time RT-PCR. §At shelter 2, 120 live cats were negative on H5 real-time RT-PCR.

The 3 H5N1 viruses isolated from dead cats in shelter 1 were designated A/feline/Korea/M302-5/2023, A/feline/Korea/M302-6/2023, and A/feline/Korea/M302-7/2023, referred to hereafter as M302-5, M302-6, and M302-7. The 4 H5N1 isolates from shelter 2 were named A/feline/Korea/M305-11/2023 (for the dead cat in the hospital), A/feline/Korea/M305-7/2023 (for the sick cat in the hospital), A/feline/Korea/M305-3/2023 and A/feline/Korea/M305-4/2023, referred to hereafter M305-11, M305-7, M305-3, and M305-4.

### Pathologic Lesions

**Figure 1:**
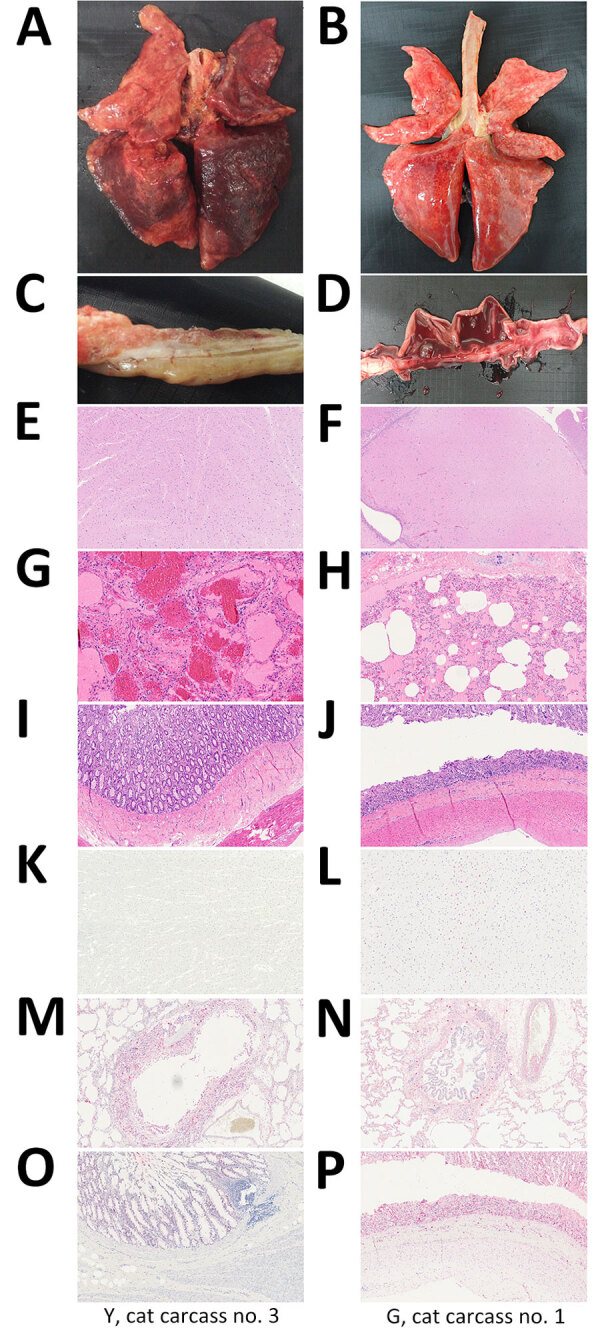
Gross, microscopic, and immunohistochemistry (IHC) findings in cats infected with highly pathogenic avian influenza A(H5N1) virus, South Korea, 2023. Findings are shown for cat carcasses from shelter 1 (Y cat carcass no. 3) and shelter 2 (G cat carcass no. 1). A–D) Gross findings: A) severe congestion and edema in the lungs; B) congestion and edema in the lungs; C) lack of lesions in the small intestine; D) bloody diarrhea in the small intestine (D). E–J) Hemotoxylin and eosin staining: E) brain showing no lesions; F) multifocal gliosis in the brain; G) interstitial pneumonia with focally extensive vascular thrombosis; H) interstitial pneumonia characterized by invasion of the alveolar lumina by mixed neutrophils and macrophages; I) intestine showing no lesions; J) necrotic enteritis with denuded villi. K–P) Immunohistochemical staining: K) brain showing no influenza virus antigens; L) influenza virus antigens in the neurons; M) influenza virus antigens in alveolar macrophages and bronchial epithelial cells; N) influenza virus antigens in alveolar macrophages and bronchial epithelial cells; O) influenza virus antigens in the small intestine; P) influenza virus antigens in the crypt epithelium and blood vessels in the submucosa. Original magnification ×100, except panel F, in which original magnification was ×10.

We first examined the carcasses (Figure 1) and assigned a body condition score (1–3, poor weight; 4–6, ideal weight; 7–9, overweight) (*23*); all 3 cat carcasses from shelter 1 (Y cat nos. 1–3) had a score of 7 (Appendix Table 1). Two carcasses (1 from shelter 1 [Y cat no. 3] and 1 from shelter 2 [G cat no. 1]) grossly exhibited diffuse moderate to severe congestion and edema in the lungs (Figure 1, panels A and B), as well as interstitial pneumonia characterized by infiltration of macrophages and degenerated neutrophils into the vascular and alveolar lumina (Figure 1, panels G and H). Y cat no. 3 did not have gross or microscopic lesions in the brain or small intestine (Figure 1, panels C, E, and I); that finding was true for all 3 carcasses from shelter 1. However, the carcass from shelter 2 had multifocal encephalitis, with gliosis and perivascular cuffing in the brain, and bloody diarrhea in the small intestine (Figure 1, panels D, F, and J). Immunohistochemistry revealed influenza virus antigens in alveolar macrophages and bronchial epithelial cells in all 4 cats (Figure 1, panels M, N). The carcass from shelter 2 also had influenza antigens in neurons, glial cells (Figure 1, panel L), and intestinal epithelial cells in the small intestine (Figure 1, panel P). No influenza viral antigens were present in the brains of carcasses from shelter 1, but H5 genes were detected (Table 1; Appendix Table 2).

### Environment Sampling

To investigate the source and extent of contamination at the cat shelters, we collected 183 environmental samples from both inside and outside the cat shelters and tested them by real-time RT-PCR. In shelter 1, we detected the M gene in samples taken from staff’s shoes and clothing, floors, doors, walls, and refrigerators. In shelter 2, we detected the M gene in vacuum cleaners and in cat feces, whereas we detected the M and H5 gene in the 4 unopened containers of raw cat food manufactured by a company using domestic duck meat as a main ingredient (Appendix Table 3).

Thereafter, we isolated H5N1 virus from the cat raw food, manufactured on July 6; we named the isolte A/environment/Korea/M305E-13/2023(H5N1), referred to hereafter as EV/M305E-13 (Table 2; Table 3). Upon conducting a nationwide recall and investigating the raw food produced by the manufacturer in question, we collected all raw food products of the same brand from customers. Of note, we isolated identical viruses not only from the same brand of food at the manufacturer but also in the food bought by customers, which was manufactured using the same lot of raw duck meat, albeit on different dates (May 26, June 15, and July 6 and 27). The level of viral infectivity in the raw food products was 10^2.5^–10^3.5^ 50% egg infectious dose (EID_50_)/g (Table 2). The viruses isolated from them were designated as A/environment/Korea/M305E2-24/2023 (M305E2-24), A/environment/Korea/M305E2-25/2023 (M305E2-25), and A/environment/Korea/M305E3-1/2023 (M305E3-1) (Table 2).

**Table 2:** Detection of H5 genes and H5 HPAIV in raw cat food in study of HPAIV A(H5N1) infection in cats, South Korea, 2023*

Sample source	Sample	Manufacturing date	H5 Ct	EID_50_/g	Strain name
Collected from shelter 2	Raw cat food† (duck meat)	2023 Jul 6	28	10^3.5^	A/environment/Korea/M305E-13/2023(H5N1)
Collected from manufacturer A‡ or the buyer of the foods under tracking investigation†	Raw cat food (duck meat)	2023 May 26	27	10^2.5^	A/environment/Korea/M305E2–24/2023(H5N1)
		2023 Jun 15	28	10^3.0^	A/environment/Korea/M305E2–25/2023(H5N1)
		2023 Jul 27	29	10^3.5^	A/environment/Korea/M305E3–1/2023(H5N1)

*Ct, cycle threshold; EID_50_, 50% egg infectious dose; HPAIV, highly pathogenic avian influenza virus. †The same brand of raw cat food products made of raw meat from ducks slaughtered on the same day but on different manufacturing dates were all positive, but the same brand of raw food products made from raw chicken meat was negative (data not shown). Moreover, all kinds of cat food made from raw meat that were recalled nationwide from 10 manufacturers tested negative except for products in Table 2. ‡Manufacturer of avian influenza–positive raw cat food product fed to cats at shelter 2.

**Table 3:** Amino acid differences identified among highly pathogenic avian influenza A (H5N1) virus detected in domestic ducks, cats, and duck meat–based cat food, South Korea, 2023*

Virus strain		Amino acid differences											
		PB2					HA†					NA‡	
Name	Origin	T271A	K526R	E627K	D701N		S137A	T160A	Q226L	G228S		H274Y	N294S
A/duck/Korea/H537/2022(H5N1)§ (EPI_ISL_18819799)	Poultry in South Korea	T	K	E	D		A	A	Q	G		H	N
A/feline/Korea/M302-5/2023(H5N1) (EPI_ISL_18819808)	Cat carcass in shelter 1	T	K	E	**N**		A	A	Q	G		H	N
A/feline/Korea/M302-6/2023(H5N1) (EPI_ISL_18819809)	Cat carcass in shelter 1	T	K	E	**N**		A	A	Q	G		H	N
A/feline/Korea/M302-7/2023(H5N1) (EPI_ISL_18819810)	Cat carcass in shelter 1	T	K	E	**N**		A	A	Q	G		H	N
A/feline/South_Korea/SNU-01/2023(H5N1) (EPI_ISL_18102700)	Cat carcass in shelter 1	T	K	E	**N**		A	A	Q	G		H	N
A/feline/South_Korea/SNU-02/2023(H5N1) (EPI_ISL_18102701)	Cat carcass in shelter 1	T	K	E	**N**		A	A	Q	G		H	N
A/feline/Korea/M305-3/2023(H5N1) (EPI_ISL_18819805)	Cat in shelter 2¶	T	K	**K**	D		A	A	Q	G		H	N
A/feline/Korea/M305-4/2023(H5N1) (EPI_ISL_18819806)	Cat in shelter 2¶	T	K	**K**	D		A	A	Q	G		H	N
A/feline/Korea/M305-7/2023(H5N1) (EPI_ISL_18819807)	Cat from shelter 2#	T	K	**K**	D		A	A	Q	G		H	N
A/feline/Korea/M305-11/2023(H5N1) (EPI_ISL_18819804)	Cat carcass from shelter 2#	T	K	**K**	D		A	A	Q	G		H	N
A/environment/Korea/M305E2-24/2023(H5N1) (EPI_ISL_18819801)	Raw duck meat food manufactured on May 26	T	K	E	D		A	A	Q	G		H	N
A/environment/Korea/M305E2-25/2023(H5N1) (EPI_ISL_18819802)	Raw duck meat food manufactured on June 15	T	K	E	D		A	A	Q	G		H	N
A/environment/Korea/M305E-13/2023(H5N1) (EPI_ISL_18819800)	Raw duck meat food manufactured on July 6	T	K	E	D		A	A	Q	G		H	N
A/environment/Korea/M305E3-1/2023(H5N1) (EPI_ISL_18819803)	Raw duck meat food manufactured on July 27	T	K	E	D		A	A	Q	G		H	N

*Letters in red denote differences between viruses. HA, hemagglutinin; NA, neuraminidase; PB2, polymerase basic 2. †H3 numbering. ‡N2 numbering. §R.M. Cha, unpub. data. ¶Cats living in shelter 2. #Cats moved from shelter 2 to the animal hospital.

We identified all facilities or companies that had handled the duck meat contained in the infectious raw cat food and tested for the presence of virus; those facilities consisted of suspected duck farms, slaughterhouses, meat processing companies, middlemen, and retailers. No virus could be detected in 214 samples from 9 locations. In addition, all types of raw foods (65 products and 24 meats from 10 manufacturers) for pet cats or dogs on the market were tested and determined to be avian influenza–negative (data not shown).

### Genetic Analysis

**Figure 2:**
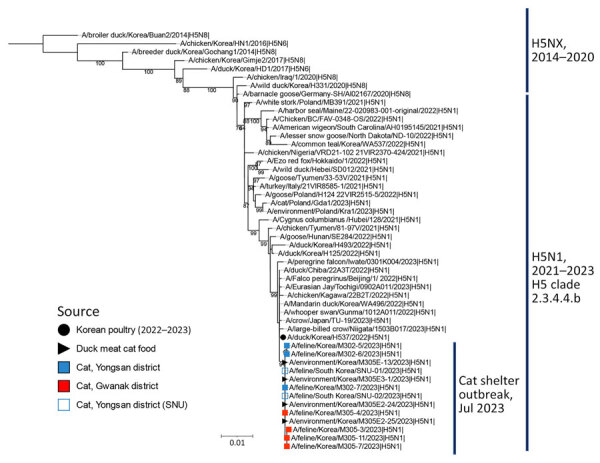
Maximum-likelihood phylogenetic tree for the hemagglutinin (HA) gene in study of highly pathogenic avian influenza virus A(H5N1) infection in cats, South Korea, 2023. The phylogenetic tree is based on H5N1 HA sequences of viruses isolated recently, as well as on the HA gene sequence of other H5Nx viruses. Bootstrap values (1,000 replicates) >70% are displayed at the branch nodes. The black circle denotes virus isolated from poultry in South Korea, 2022–2023, and the black triangle denotes viruses isolated from raw duck meat used for cat food. The blue shaded square denotes viruses isolated from cats in shelter 1, and the red square indicates viruses isolated from cats in shelter 2. The blue outlined square indicates viruses isolated from cats in shelter 1 by SNU. Scale bar indicates number of nucleotide substitutions per site. SNU, Seoul National University.

**Figure 3:**
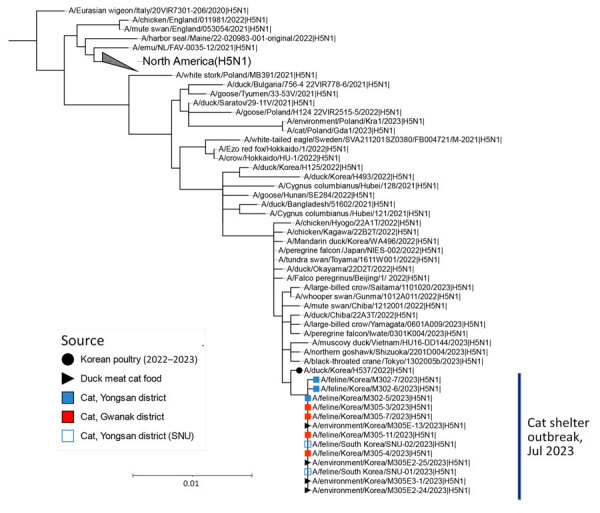
Maximum-likelihood phylogenetic tree for the neuraminidase (NA) gene in study of highly pathogenic avian influenza virus A(H5N1) infection in cats, South Korea, 2023. The phylogenetic tree is based on sequences of H5N1 NA sequences of viruses isolated recently, as well as on the HA gene sequence of other H5Nx viruses. Bootstrap values (1,000 replicates) >70% are displayed at the branch nodes. The black circle denotes virus isolated from poultry in South Korea, 2022–2023, and the black triangle denotes viruses isolated from raw duck meat used for cat food. The blue shaded square denotes viruses isolated from cats in shelter 1, and the red square indicates viruses isolated from cats in shelter 2. The blue outlined square indicates viruses isolated from cats in shelter 1 by SNU. Scale bar indicates number of nucleotide substitutions per site. SNU, Seoul National University.

To identify the source of the H5N1 virus isolated from cats and raw food, we analyzed representative viruses isolated at each location alongside other viruses within H5Nx clade 2.3.4.4b (Figures 2, 3). The 8 genes of the 3 viruses, M302-5, M305-11, and M305E-13, were almost identical among them (99.9% for nucleoprotein, HA, and PB1 and 100% for polymerase acidic [PA], NA, M, and nonstructural [NS]). Phylogenetic analysis revealed that the HA and NA genes of the 3 viruses are most closely related to those of the H5N1 clade 2.3.4.4b identified in 2022–2023 (Figures 2, 3), 1 of which was A/duck/Korea/H537/2022(H5N1); nucleotide identities to them were 99.46%–100% across the 8 genes (Appendix Table 4). All the cat viruses isolated from shelter 1, including M302-5, possessed mutation D701N in the PB2 gene, whereas all the cat viruses isolated from shelter 2, including M305-11, possessed mutation E627K in the PB2 gene (Table 3; Appendix Table 4). The D701N and E627K mutations in the PB2 gene are critical markers of virus adaptation to mammals (*12*).

## Discussion

The owner of shelter 1 in Seoul, in which 38 of 40 cats died within a month beginning in late June 2023 (*15*), had taken sick cats with respiratory and neurologic symptoms to a private animal hospital in early July. Of the 38 cats that died, HPAIV was diagnosed in only 5; the other 33 cat carcasses had been disposed of without diagnosis (*15*). After the report of the HPAI case in shelter 1, all cat owners, shelters, and veterinarians were urged to report influenza-like illnesses to the government, and another suspicious case in cats originating from shelter 2 was disclosed.

The HPAI infections of dogs (2015) and cats (2016) previously reported in South Korea were related to infected wild birds (*13*,*14*). However, the infections of cats in 2 shelters located in a metropolitan city in 2023 could not be attributed to direct contact with wild birds or poultry. Therefore, the HPAI-contaminated raw cat food found at shelter 2 was regarded as a critical and direct source of infection. Furthermore, the viruses isolated from the cats (M305-11) and the raw food from shelter 2 (M305E-13) presented a genetic similarity >99.9% for all the genes, and that similarity strongly supports the idea that the raw food was the direct source of infection, particularly in shelter 2. The gene constellation of M302-4, M305-11, and M305E-13 was most similar to that of viruses isolated in Korea in November 2022; 1 of those was A/duck/Korea/H537/2022(H5N1), which had a nucleotide identity of 99.46%–100% across the 8 genes (Appendix Table 4). These viruses possess a gene constellation representing that of a group, including A/duck/Korea/H537/2022(H5N1), dominant in South Korea (R.M. Cha, unpub. data) in the winter of 2022–2023, which was also isolated from birds in Japan during the same period (*15*).

The viral infectivity of the contaminated raw food product ranged from 10^2.5^ to 10^3.5^ EID_50_/g, which is similar to the minimal dose required to infect cats (10^2^–10^4^ EID_50_/g) (*24*). The high viral load in most organs from the dead cats suggests that the virus replicated systemically and affected the host severely, similar to the effects of HPAI in chickens. The pattern of distribution of viral load, virus particles, and lesions observed in the cat carcasses was very similar to that observed in other HPAI H5N1–infected cats (both naturally and experimentally infected cases) (*24*). Previous studies report that gastrointestinal exposure is sufficient to infect cats with HPAIV; the liver and lungs are the main organs affected (*25*,*26*). We also found that the liver, lungs, and especially the intestines of cats from shelter 2 had the highest viral load among all organs (cycle threshold 12–19), along with clear pathologic lesions (Figure 1). Our results support the fact that oral consumption of contaminated raw food products can induce extensive lesions in the digestive system, along with concurrent infection of the respiratory and digestive systems (Figure 1) (*27*). The dead cats, and those with clinical signs, at shelter 2 had likely ingested the cat raw food repeatedly, resulting in substantial exposure to the virus. By contrast, the cat carcasses from shelter 1 had been stored in a frozen state, making it difficult to determine the route of HPAI infection on the basis of pathologic lesions alone. Although no direct evidence of contaminated food was found at shelter 1, the cause of infection is presumed to be the same as at shelter 2; that presumption is based on a statement from the owner of shelter 1 that cats had been fed a variety of types of raw food and the discovery of a receipt for the purchase of the same brand of raw cat food consumed in shelter 2. Of note, the 2 kinds of mutations related to mammalian adaptations, PB2-E627K and PB2-D701N, were observed in the viruses isolated from cats in shelter 1 (PB2-E627K) and shelter 2 (PB2-D701N). However, in the viruses isolated from the raw cat food, none of these point mutations were observed. The mammal-adaptive mutations at the critical genome sites of the HPAI virus are the same as those reported previously (*28*,*29*).

In other genomic regions, the viruses isolated from the cats in shelter 2 had amino acid differences in a few locations (Appendix Table 5). Each of those cat viruses in shelter 2, notably, had quasispecies containing minor populations with glutamic acid (E) at 627th in PB2 and major populations with lysine (K) at the same location (data not shown). Therefore, in the case of shelter 2, all the cats were likely infected from the direct ingestion of the contaminated raw foods. For the cat viruses from shelter 1, most of the deceased cats and raw food were disposed of before testing, and feeding records for the infected animals were not maintained, making the route of infection and transmission in that shelter difficult to infer. HPAI infection of cats in Poland during the summer of 2023 was suspected to be caused primarily by cat food made from poultry meat (*11*,*12*).

The main ingredient of the raw food collected from shelter 2 was domestic duck meat, and we suspect some infected broiler ducks were slaughtered despite intensive and regular active avian influenza surveillance on broiler duck farms during the HPAI incursion period; broiler duck farms should be tested 3–4 times for avian influenza before ducks are moved to the slaughterhouse (*30*). Further epidemiologic investigations revealed that the cat food manufacturer had not performed the required electron beam sterilization process during production, and the omission of the sterilization process is considered the most direct cause of cat infection in the shelters (data not shown). Thus, all facilities or companies that handled duck meat were contacted and ordered to clean and disinfect the premises to prevent secondary infections by avian influenza viruses. Promptly identifying the source of infection in shelter 2 led to the recall of all contaminated raw cat food products or products at risk for contamination; all were discarded.

The cases of HPAI infection at 2 cat shelters caring primarily for stray cats located in Seoul, South Korea, were sporadic and irregular. The source of infection at shelters was improperly sterilized raw cat food. We identified systemic virus and pathologic manifestations in the carcasses of cats that had consumed this raw food and confirmed the presence of mammalian-adaptive mutations in the viruses isolated from the cats. From these results, ways to increase disease surveillance sensitivity on poultry farms continue to be sought on the basis of the risk-based surveillance principle (*31*). In addition, more strict disease monitoring in the slaughterhouse is also necessary, especially for subclinical infection of duck species. As a last resort, the risk for avian influenza virus infection in pets should be mitigated by achieving compliance and enforcing regulations for sterilization of raw food. Moreover, it was also perceived that humans exposed to the risk for HPAI infection must be identified and monitored, and various preventive measures have been implemented by the authorities for human health. In conclusion, strict management and adequate sterilization for raw poultry meat are required, along with active surveillance, to prevent influenza-like illnesses that could become a public health concern.

Appendix Additional information about highly pathogenic avian influenza A(H5N1) virus infection in cats, South Korea, 2023
