# Supplementary material for: Highly Pathogenic Avian Influenza A(H5N1) Virus Infection in Cats, South Korea, 2023
Source: Emerg Infect Dis. 2024 Dec;30(12):2510–20. doi: 10.3201/eid3012.240154 (PMC11616667; doi:10.3201/eid3012.240154)
Supplement: Appendix — Additional information about highly pathogenic avian influenza A(H5N1) virus infection in cats, South Korea, 2023 [file 24-0154-Techapp-s1.pdf]

Article DOI: https://doi.org/10.3201/eid3012.240154

EID cannot ensure accessibility for supplementary materials supplied by authors. Readers who have difficulty accessing supplementary content should contact the authors for assistance.

Highly Pathogenic Avian Influenza A(H5N1) Virus Infection in Cats, South Korea, 2023

Appendix.

Appendix Table 1. Gross lesions in various tissues of four cats diagnosed with highly pathogenic avian influenza, South Korea

Tissue	Lesions	Y- Cat No.1*	Y-Cat No.2*	Y-Cat No.3*	G-Cat No.1*
Body	Body Condition Score	7	7	7	5
Eye	Jaundice	none	mild	moderate	severe
Trachea	Congestion	mild	mild	mild	mild
Lung	Edema, Congestion, hemorrhage	severe	severe	severe	severe
Heart	Pericardial effusion, hemorrhage	moderate	moderate	moderate	moderate
Spleen	Enlarged, hemorrhage	none	severe	moderate	moderate
Liver	Necrotic foci	severe	severe	severe	severe
Intestine	Hemorrhage, bloody contents	none	None	None	severe

*Y-Cat No.1, Cat carcass No.1 in Shelter 1; Y-Cat No.2, Cat carcass No.2 1 in Shelter 1; Y-Cat No.3, Cat carcass No.3 in Shelter 1; G-Cat No.1, Cat carcass No.1 in Shelter 2.

Appendix Table 2. Pathologic lesions and reactivity to anti-influenza viral nucleoprotein in various tissues of four cats diagnosed with high pathogenicity avian influenza, South Korea, 2023*

	Hematoxylin and eosin stain					Immunohistochemical stain				
Tissue	Lesions	Y-Cat No.1	Y-Cat No.2	Y-Cat No.3	G-Cat No.1	Cells	Y-Cat No.1	Y-Cat No.2	Y-Cat No.3	G-Cat No.1
Brain	Neuronal Necrosis<br>Meningoencephalitis<br>Gliosis	none<br>none<br>none	none<br>none<br>none	none<br>none<br>none	Moderate<br>Moderate<br>Moderate	Neurons<br>Ependymal cells<br>Glial cells	none<br>none<br>none	none<br>none<br>none	none<br>none<br>none	Moderate<br>Moderate<br>Moderate
Lung	Interstitial pneumonia<br>Pneumocytic necrosis	Severe<br>Severe	Severe<br>Severe	Severe<br>Severe	Moderate<br>Moderate	Macrophages<br>Bronchial epithelial cells	Severe<br>Severe	Severe<br>Severe	Severe<br>Severe	Moderate<br>Moderate
Heart	Myocytic necrosis	Mild	Mild	Mild	Mild	Myocytes	Mild	Mild	Mild	Mild
Spleen	Lymphocytic necrosis<br>Lymphocytic depletion	Mild<br>Mild	Moderate<br>Moderate	Mild<br>Mild	Mild<br>Mild	Macrophages & necrotic debris	Mild	Mild	Mild	Mild
Liver	Hepatic necrosis	Severe	Severe	Severe	Severe	Kupffer cells and necrotic debris	Severe	Severe	Severe	Severe

*Y-Cat 1, Cat carcass No.1 in Shelter 1; Y-Cat 2, Cat carcass No.2 1 in Shelter 1; Y-Cat 3, Cat carcass No.3 in Shelter 1; G-Cat 1, Cat carcass No.1 in Shelter 2.

Appendix Table 3. Molecular detection of avian influenza in environmental samples from the cat shelters at Shelter 1 and Shelter 2

Working zone		Sampling site	Number of samples	M-positive samples	H5-positive samples
Shelter 1	Inside	Cage	8	0	0
		Human shoes or clothes	2	1	0
		Feces of cats	3	0	0
		Floor, door, wall	40	2	0
		Refrigerator	2	1	0
		Vehicle	2	0	0
		Dried food	11	0	0
	Outside	Feathers of wild birds	4	0	0
		Feces of wild birds	2	0	0
Shelter 2	Inside	Cage	18	0	0
		Cat material	10	0	0
		Feces of cats	12	2	0
		Dried food, water	28	0	0
		Raw meat food (chicken meat)	22	0	0
		Raw meat food (duck meat)	4	4	4
		Floor, door, wall	12	0	0
		Human shoes or clothes	10	0	0
		Vacuum cleaner	10	1	0
		Window	4	0	0
	Outside	Vehicle	2	0	0
		Feathers of wild birds	10	0	0
		Feces of wild birds	4	0	0
Total			183	11	4

Appendix Table 4. Nucleotide identity versus influenza A/environment/Korea/AM305E-13/2023(H5N1) isolated from duck meat cat food

No.	Strain	Origin	PB2	PB1	PA	HA	NP	NA	M	NS
1	A/duck/Korea/H537/2022(H5N1)	Korean Poultry	99.77	99.6	99.58	99.71	99.53	99.79	99.9	100
2	A/feline/Korea/M302–5/2023(H5N1)	Cat carcass in Shelter 1	99.95	99.91	100	99.94	100	100	100	100
3	A/feline/Korea/M302–6/2023(H5N1)	Cat carcass in Shelter 1	99.91	99.91	100	99.94	100	99.93	100	100
4	A/feline/Korea/M302–7/2023(H5N1)	Cat carcass in Shelter 1	99.95	99.91	99.95	100	100	99.93	100	100
5	A/feline/South_Korea/SNU-01/2023(H5N1)	Cat carcass in Shelter 1	99.95	-	-	100	-	99.93	100	100
6	A/feline/South_Korea/SNU-02/2023(H5N1)	Cat carcass in Shelter 1	99.91	-	-	100	-	99.93	100	100
7	A/feline/Korea/M305–3/2023(H5N1)	Cat in Shelter 2	99.95	100	99.95	99.88	99.93	100	100	100
8	A/feline/Korea/M305–4/2023(H5N1)	Cat in Shelter 2	99.95	100	100	100	100	100	100	100
9	A/feline/Korea/M305–7/2023(H5N1)	Cat from Shelter 2	99.95	100	100	99.94	99.93	100	100	100
10	A/feline/Korea/M305–11/2023(H5N1)	Cat carcass from Shelter 2	99.95	100	100	99.94	99.93	100	100	100
11	A/environment/Korea/M305E2–24/2023(H5N1)	Raw duck meat food manufactured on 26th May	100	100	100	100	100	100	100	100
12	A/environment/Korea/M305E2–25/2023(H5N1)	Raw duck meat food manufactured on 15th June	100	100	100	100	100	100	100	100
13	A/environment/Korea/M305E3–1/2023(H5N1)	Raw duck meat food manufactured on 27th July	100	100	100	100	100	100	100	100

Appendix Table 5. Additional amino acid differences identified among influenza A (H5N1) virus detected in Korean domestic ducks, cats, and duck meat-based cat food, Korea*

Virus strain		PB2		PB1				PA	HA	HA
Name	Origin	I3M	P104T	K52R	G211R	Q460K	E752G	V669I	D99N	S519G
A/duck/Korea/H537/2022(H5N1)	Korean Poultry	I	P	K	G	Q	E	V	D	S
A/feline/Korea/M302–5/2023(H5N1)		M	P	R	R	Q	E	V	D	S

Virus strain		PB2		PB1				PA	HA	HA
Name	Origin	I3M	P104T	K52R	G211R	Q460K	E752G	V669I	D99N	S519G
A/feline/Korea/M302–6/2023(H5N1)	Cat carcass in shelter 1	I	T	K	R	Q	G	V	D	S
A/feline/Korea/M302–7/2023(H5N1)		M	P	K	R	K	E	V	D	S
A/feline/South_Korea/SNU-01/2023(H5N1)		N/A	P	K	G	Q	E	N.A.	D	S
A/feline/South_Korea/SNU-02/2023(H5N1)		N/A	P	K	G	Q	E	N.A.	D	S
A/feline/Korea/M305–3/2023(H5N1)	Cats in shelter 2	I	P	K	G	Q	E	I	N	G
A/feline/Korea/M305–4/2023(H5N1)		I	P	K	G	Q	E	V	D	S
A/feline/Korea/M305–7/2023(H5N1)		I	P	K	G	Q	E	V	N	S
A/feline/Korea/M305–11/2023(H5N1)	Cat carcass from shelter 2	I	P	K	G	Q	E	V	N	S
A/environment/Korea/M305E2–24/2023(H5N1)	Raw duck meat food manufactured on 26th May	I	P	K	G	Q	E	V	D	S
A/environment/Korea/M305E2–25/2023(H5N1)	Raw duck meat food manufactured on 15th June	I	P	K	G	Q	E	V	D	S
A/environment/Korea/M305E-13/2023(H5N1)	Raw duck meat food manufactured on 6th July	I	P	K	G	Q	E	V	D	S
A/environment/Korea/M305E3–1/2023(H5N1)	Raw duck meat food manufactured on 27th July	I	P	K	G	Q	E	V	D	S

*N/A, not available
